# Intelligence and Dietary Habits: An International Study of Mensa Members

**DOI:** 10.3390/jintelligence13060067

**Published:** 2025-06-10

**Authors:** Anna Csák, Péter Przemyslaw Ujma

**Affiliations:** 1 Health Science Division, Doctoral College, Semmelweis University, 1085 Budapest, Hungary; 2 Institute of Behavioural Sciences, Semmelweis University, 1089 Budapest, Hungary

**Keywords:** dietary habits, lifestyle, diet, Mensa, giftedness

## Abstract

Numerous studies have shown a positive relationship between intelligence and health, with higher intelligence quotient (IQ) linked to better health outcomes, longer life expectancy, and lower rates of non-communicable diseases. Better health behaviour in the more intelligent (either due to better health knowledge or more advantageous social-financial opportunities) and system integrity theory (overlaps in the background causes of intelligence and health, such as genetic factors) are competing explanations for this link. This study aimed to examine the dietary habits of high-IQ individuals compared to a control group. An online questionnaire was completed by Mensa members (IQ ≥ 130) and control group participants from three countries, assessing various lifestyle factors, especially dietary habits. Key findings include lower smoking rates among Mensa members, special diets primarily for personal rather than medical reasons, and more frequent consumption of some national staples. There was no clear trend for healthier nutritional habits among Mensa members, suggesting that this aspect of health behavior does not account for better health in the more intelligent and supporting system integrity theory instead.

## 1. Introduction

A healthy lifestyle is built on several pillars, with physical activity, good nutrition (World Health Organization 2010), mental health (World Health Organization 2024b), and adequate fluid intake (Institute of Medicine 2005) being key elements. Health behaviour, which affects these, is influenced by several factors, both biological and non-biological (Braveman et al. 2011). The association of these factors with health status has been extensively studied with modern designs enabling the separation of putative causal effects from simple correlations. There is a positive longitudinal association between intelligence and health (Fries and Pietschnig 2022), with higher intelligence quotient (IQ) being associated with higher life expectancy and lower prevalence of non-communicable, civilizational diseases (Batty et al. 2009; Calvin et al. 2017; Christensen et al. 2016). Studies have shown that life expectancy increases with higher childhood IQ scores (Fries and Pietschnig 2022; Whalley 2001), and this phenomenon is also observed when controlling for socioeconomic status (SES) (Der et al. 2009). Cross-sectional correlational studies are used to investigate the association of factors classified as non-medical factors, such as personal characteristics (education, income, and health-related attitudes), microenvironment (family, work environment, and friends), and macroenvironment (cultural characteristics of the area, food availability, and economic situation) (McKinlay 2023) with health. Advanced research designs do not always confirm the causal role of psychosocial factors associated with better health. For example, when comparing monozygotic twin pairs discordant for education or social status to account for common familial causes of SES and health, negative findings are common with higher SES members not having better health (Amin et al. 2018; Fujiwara and Kawachi 2009; Krieger et al. 2005). Pseudo-experimental studies using Mendelian randomization and regression discontinuity analysis centered on education reforms in Britain (Clark and Royer 2010) found some putative causal effects of education but confirmed that most of the association between SES and health is not direct but likely relies on shared causes. The above results suggest that the effect of SES on health is likely lower than it appears in correlational studies.

Intelligence may be another crucial factor for health (Gottfredson and Deary 2004), with competing theories assuming either direct effects or common causes, as with SES (Deary et al. 2021). A direct effect may be through health literacy, which refers to the fact that high levels of cognitive ability can be utilized to understand health-related information as well as to plan and maintain health-preserving life strategies (Gottfredson 1998; Neisser et al. 1996; Gottfredson 2004). Another theory about direct effects assumes that better adult life conditions, such as higher income or having a more prestigious job, are enabled by higher intelligence (Herrnstein and Murray 1994; Strenze 2007), and these can also contribute to achieving and maintaining health. Better time management, better working conditions, and financial security provide opportunities to ensure conditions that support health (e.g., exercise and good-quality food) (Barakat and Konstantinidis 2023). These theories would predict better health behaviours in those with higher intelligence, which mediates the link to lower morbidity and mortality. The main common causes theory is system integrity (Deary et al. 2021; Deary 2012), which assumes that a likely genetic predisposition for having generally higher health leads to both higher IQ and, subsequently, better health (Deary 2012). The lack of a link between intelligence and health behaviour would be in line with this hypothesis, as, in this case, better health is not a consequence of the lifestyle enabled by higher intelligence.

Research does not consistently show a positive association between health behaviour and intelligence. Although a positive relationship is observed for smoking, with less frequent use typical among individuals with higher IQ (Hemmingsson et al. 2008; Kubicka et al. 2001; Batty et al. 2007b), in the case of certain nutrition-related behaviours (e.g., alcohol consumption, reading product labels, meal regularity, skipping meals, and snacking between main meals), higher IQ does not correlate with better health behaviours (Calvin et al. 2017; Christensen et al. 2016; Kanazawa and Hellberg 2010; Belasen and Hafer 2013). Good nutrition is also associated with better health (Przybyłowicz and Danielewicz 2022), so people with good nutrition can be expected to have better health (Shan et al. 2020). Good nutrition improves the body’s resistance to disease and reduces the incidence of chronic disease (Koch et al. 2024). The relationship between intelligence and health behaviour is often investigated, but research on the relationship between IQ and nutrition is scarce and has been predominantly conducted in children (Ghazi et al. 2013; El-Kholy and Elsayed 2015). Studies have operationalized dietary assessment by measuring the frequency of consumption of certain foods (often without specifying the amount), using this as a primary determinant of overall diet (Wang et al. 2021; Khadem et al. 2024) over different intervals. Wang et al. (2021) investigated the relationship between dietary patterns and cognitive abilities in Chinese children; however, the study used the frequency of food consumption as the primary metric for diet, which does not always accurately reflect diet quality. Similarly, Khadem et al. (2024) analysed the impact of dietary habits and physical activity on the intelligence of primary schoolchildren, but their findings were more indicative of general trends rather than specific mechanisms. Based on the above, it is evident that while measuring the frequency of food consumption is common in studies of dietary habits, a comprehensive assessment of diet requires more precise quantification of intake.

The aim of our study is not to re-establish or causally explain the well-documented link between intelligence and health, but rather to examine whether distinctive, consistent patterns of dietary behavior can be observed among individuals with high intelligence. To this end, we compared the detailed dietary habits—including both frequency and quantity of consumption—of objectively high-IQ individuals from three countries (Hungary, Germany, and United Kingdom) to an international control group. This dual-level assessment provides a foundation for understanding how cognitive ability may manifest in everyday lifestyle choices, irrespective of whether these behaviours cause or result from better health outcomes.

## 2. Materials and Methods

Our study was based on an international online survey administered to participants from a high-IQ society (Mensa International) and control participants in multiple countries. The survey aimed to assess the lifestyle of the participants, with a particular focus on dietary habits.

### 2.1. Population

The highly intelligent participants were members of the Mensa International association. This international organisation brings together people with an IQ of 130 or above, as measured by their test. Participants were contacted through local organisations in the three countries (Hungary, Germany, and United Kingdom), and the questionnaire was published only on platforms accessible to them (e.g., mailing lists, official websites, and online closed groups). Control persons were engaged via online social media platforms. The link to the questionnaire was also available in thematic research groups and websites (SurveyCircle and Facebook), as well as in groups of people living in the area (e.g., in United Kingdom/Germany). Due to the nature of these platforms, it was expected that a younger audience would be reached who were active online. Persons who incompletely completed the mandatory parts of the questionnaire and those who had undergone previous digestive tract surgery were excluded (*n* = 106).

### 2.2. Demographics

Before the questions on dietary habits, participants were asked to complete the section on demographics and lifestyle. The self-reported variables are shown in Supplementary Table S1.

### 2.3. Dietary Intake

**Figure 1:**
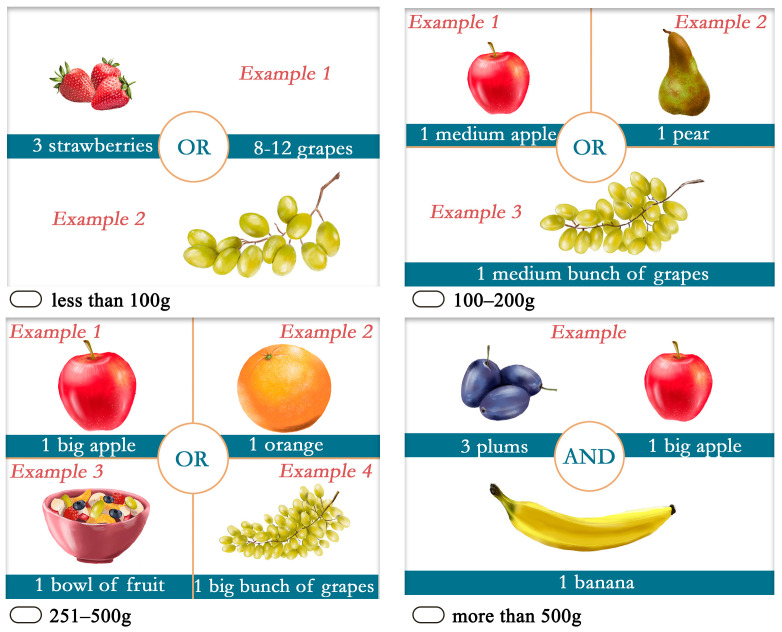
Examples of portion sizes to help answer the question “How much fruit do you eat per meal?”.

Participants completed a self-developed, semi-quantitative food frequency questionnaire (SQ-FFQ) to assess their eating habits. The questionnaire consisted of a list of 70 food items and asked respondents to report on the frequency with which they consume the listed items. These questions were categorical, with the following response options: “many times a day,” “once a day,” “more than 6 times a week but not daily,” “4–6 times a week,” “1–3 times a week,” “6–8 times a month but not weekly,” “4–5 times a month but not weekly,” “1–3 times a month,” “less often,” and “never,” except for dairy products, where the response options were these: “many times a day,” “once a day,” “more than 6 times a week but not daily,” “4–6 times a week,” “1–3 times a week,” “1–5 times a month but not weekly,” “less often,” and “never.” For 35 foods, they also had to indicate the amount of each food consumed in a meal. The section of the questionnaire aimed at assessing the frequency of consumption and intake amounts for each commodity is included in Supplementary Table S2. The definition of the portion sizes was also facilitated by visual (portion size pictures—Figure 1) and textual (e.g., “a small cup of yoghurt,” “a tablespoon,” “approx. two tablespoons,” or “a medium slice”) information.

The monthly intake of each ingredient was calculated by multiplying the frequency of consumption by the weight of servings consumed per session. For consumption, frequencies and portion sizes reported as interval categories (e.g., “1–3 times a month” or “101–150 g”) and the interval midpoint (e.g., “2 times a month” or “125 g”) were used for calculations. For some raw materials, the unit sizes of the products have been adapted to the range of products available in the country. To standardize responses and ensure consistent quantification, all quantity-related items were presented as closed-ended interval categories (e.g., “less than 50 g” or “101–150 g”) without free-text options. These categories were designed based on the authors’ practical experience, international portion size conventions, and commonly available product packaging in Hungary, Germany, and the UK. Wherever applicable, estimates were informed by average sizes of everyday food items (e.g., “one tablespoon of sour cream” or “≈20 g”). This structured design minimized variability due to respondent interpretation, enabling reliable conversion of reported portions into grams for statistical analysis.

Inspection of the data revealed a set of extreme values (e.g., dozens of kilograms of monthly oilseeds consumption), likely resulting from misunderstanding the scale of measurement. In order to eliminate the effect of such extreme values, we deleted outliers beyond 2 SD above the mean value. Using a 3 SD cutoff did not change results substantially (see Section 3).

The following variables were asked about nutrition-related behaviour:

- Keeping a food diary (response options: “yes” or “no”). Persons who regularly kept a food diary could indicate the interface (response options: “in application,” “written on paper or in a document,” or “other”) and reason for monitoring their meals (response options: “calorie counting,” “systematization,” “carbohydrate counting,” or “other”).
- Following a special diet (answer options: “gluten-free,” “lactose-free,” “dairy-free,” “sugar-free,” or “other”) and the reason for this (answer options: “medical advice,” “diagnosis,” “personal choice,” or “other”).

Healthy dietary habits were considered to include regular and abundant consumption of vegetables and fruits, a preference for whole grains over low-fiber alternatives, frequent consumption of fish and seafood, and regular intake of oilseeds and legumes. Additionally, moderate meat consumption, low consumption of processed meat products, and regular intake of low-fat and plain dairy products were considered part of healthy dietary habits. Maintaining moderate caffeine intake (fewer than 4 cups of coffee per day) was also included. Furthermore, we considered it a positive factor if participants followed a special diet based on sound reasons, such as medical recommendations or scientifically supported health benefits, rather than decisions based solely on personal preferences. Unhealthy dietary habits were classified as low consumption of vegetables and fruits, rare consumption of fish and seafood, occasional and minimal intake of nuts and legumes, and regular consumption of large amounts of meat. Frequent and high consumption of processed meat products, regular consumption of high-fat and flavored dairy products, high caffeine intake (more than 4 cups of coffee per day), and excessive alcohol consumption were also considered unhealthy habits.

### 2.4. Analytical Strategy

In our analyses, we aimed to discover food consumption patterns, which are typical in the high-IQ population compared to controls. In order to establish this, in three separate models, we compared self-reported consumption in the international control population to the Hungarian, British, and German Mensa members for each dietary item with simple OLS regressions implemented in the fitlm() MATLAB 2022a function. Due to group differences in sex composition and age (see Section 3), we used these demographic variables as covariates in addition to the binary grouping variable used as the main predictor of interest.

For items where exact quantities (e.g., grams) could be calculated, we used ordinary linear models, and the effect sizes are group differences in consumed quantities. For binary items (e.g., smoking), we used logistic regression, and the effect sizes are odds ratios.

Due to the large number of tests (35 food items, 3 groups), we applied two strategies to avoid spurious findings. First, we used the Benjamini–Hochberg method (Benjamini and Hochberg 1995) to correct for false discovery rates resulting from multiple testing applied across all food items for each of the three subgroups. Second, by using three separate high-IQ populations sampled from three different countries, we ranked findings by across-country replication frequency and consequently by strength of confidence in the following way:

#### 2.4.1. Across-Country Replications

We put the highest confidence in findings, which replicated across countries, suggesting a general difference in food consumption in high IQ compared to control populations. We considered a nutrition trend to replicate across countries if the direction of effects was concordant across all countries and reached corrected statistical significance in at least two.

#### 2.4.2. Partial Across-Country Replications

High confidence was placed in findings, which showed some concordance across countries. We considered a nutrition trend to be partially replicated across countries if the direction of effects was concordant across all countries and reached corrected statistical significance in at least one country and uncorrected significance in at least another.

#### 2.4.3. Country-Specific Findings

Some nutritional trends in the high-IQ population might be unique to a certain country due to cultural reasons: for example, high-IQ individuals may forego consuming a food item considered unhealthy, which is otherwise commonly consumed in that particular culture. For this reason, we also considered findings that were observed only in a single high-IQ population as a possible country-specific trend, as long as it reached significance after correcting for multiple testing. We also considered findings to be country-specific if they were significant in multiple countries with opposite signs.

In order to avoid false positives, we did not consider findings not passing correction for multiple testing by themselves, even if they showed some across-country concordance. We conducted additional analyses to examine whether differences in educational attainment may have confounded the observed associations. Participants reported their education as a categorical variable, which we transformed into years of education as follows: 10 years (less than high school), 12 years (high school), 15 years (bachelor’s degree), 17 years (master’s degree), and 21 years (doctoral degree). These values were included as an additional covariate in supplementary analyses to evaluate whether years in education influenced the relationship between Mensa membership and dietary habits.

## 3. Results

### 3.1. Demographics

A total of 587 Mensa participants responded to the survey. Of these, 190 participants were from Hungary (HU), 257 from Germany (DE), and 140 from the United Kingdom (UK). A total of 317 Mensa members were female and 264 were male, while 6 chose an “other” option.

We collected 196 responses from control participants. Of these, 114 were from Hungary, 24 from United Kingdom, and 12 from the United States, while the rest were from 26 various countries, with a maximum of 5 respondents each. A total of 144 control participants were female and 52 male, with 0 “other” responses.

The mean age of the sample was 44.33 years (SD = 15.33 years). Control participants were significantly younger than Mensa members (Mean_mensa_ = 48.82 years, SD = 13.87 years; Mean_control_ = 30.90 years, SD = 11.05 years; *p* < 0.001). Due to age and sex differences between Mensa members and control participants, all models were controlled for age and sex.

Detailed descriptive statistics are available in Supplementary Table S3. Detailed statistics for all variables are reported in Supplementary Table S4.

### 3.2. Across-Country Replications

**Figure 2:**
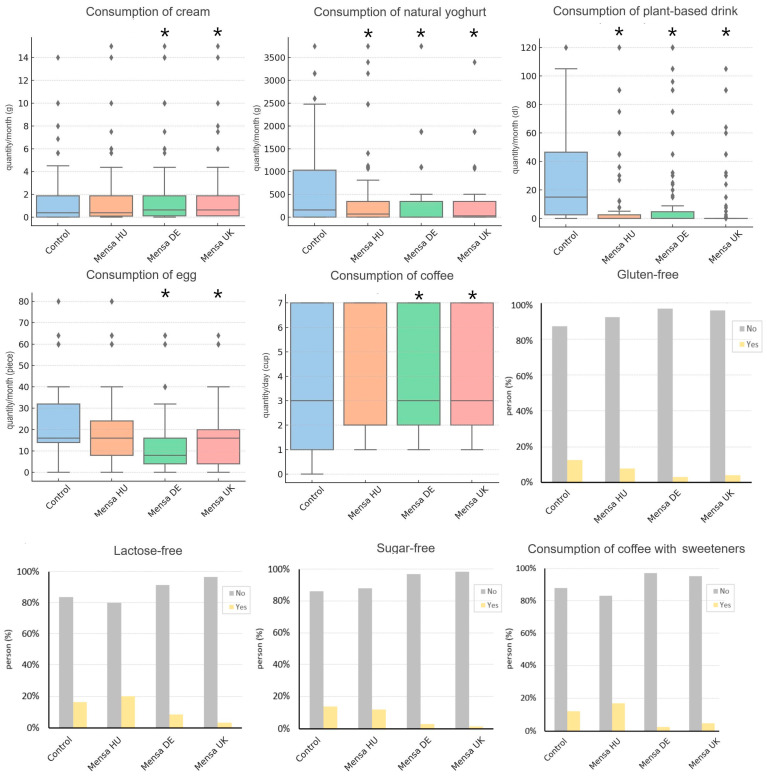
Across-country replications among Mensa members in Hungary (HU), Germany (DE), and United Kingdom (UK). Asterisks indicate statistically significant differences (*p* < 0.05) compared to the control group. Diamonds indicate outliers.

We considered a nutrition trend to replicate across countries if the direction of effects was concordant across countries and reached corrected statistical significance in at least two. Mensa members in all three countries were less likely to consume gluten-free products (OR = 0.11–0.33), and the consumption of lactose-free (OR = 0.19–0.35) and sugar-free (OR = 0.05–0.53) ingredients was less frequent in the German and British Mensa subsamples. Although the Hungarian Mensa members also had less frequent consumption (OR_lactose-free_ = 0.98; OR_sugar-free_ = 0.53), their results were not statistically significant (p_lactose-free_ = 0.95; p_sugar-free_ = 0.073). Among dairy products, Mensa members in all three countries had a higher intake of animal cream (B_HU_ = 0.34 g/month, B_DE_ = 1.05 g/month, B_UK_ = 1.19 g/month, significant in DE and UK) and a lower intake of natural yoghurt (B_HU_ = −259.68 g/month, B_DE_ = −400.20 g/month, B_UK_ = −485.97 g/month, corrected significant in all three countries). Across all countries, lower consumption of plant-based drinks (B_HU_ = −14.27 g/month, B_DE_ = −13.70 g/month, B_UK_ = −13.80 g/month, significant in all (p_HU_ = 3 × 10^−5^, p_DE =_ 4.4 × 10^−5^, p_UK =_ 9.1 × 10^−4^)) and eggs (B_DE_ = −12.20 piece/month, B_UK_ = −9.49 piece/month, B_HU_ = −4.31 piece/month, significant in B_DE and_ B_UK_) was found. Daily coffee consumption was observed with higher intakes in all three countries (B_HU_ = 0.19 cup/day, B_DE_ = 0.74 cup/day, B_UK_ = 0.83 cup/day), which were significant in the German (*p* = 0.003) and English (*p* = 0.013) Mensa subsamples. In the latter two subsamples, coffee consumption with sweeteners was lower (OR_DE_ = 0.16; OR_UK_ = 0.23), which remained significant after correction (pDE = 0.0001; pUK = 0.008). However, among Hungarian Mensa members, a higher intake was measured (OR = 1.21; *p* = 0.55) compared to the control group. Figure 2 illustrates these findings.

### 3.3. Partial Across-Country Replications

**Figure 3:**
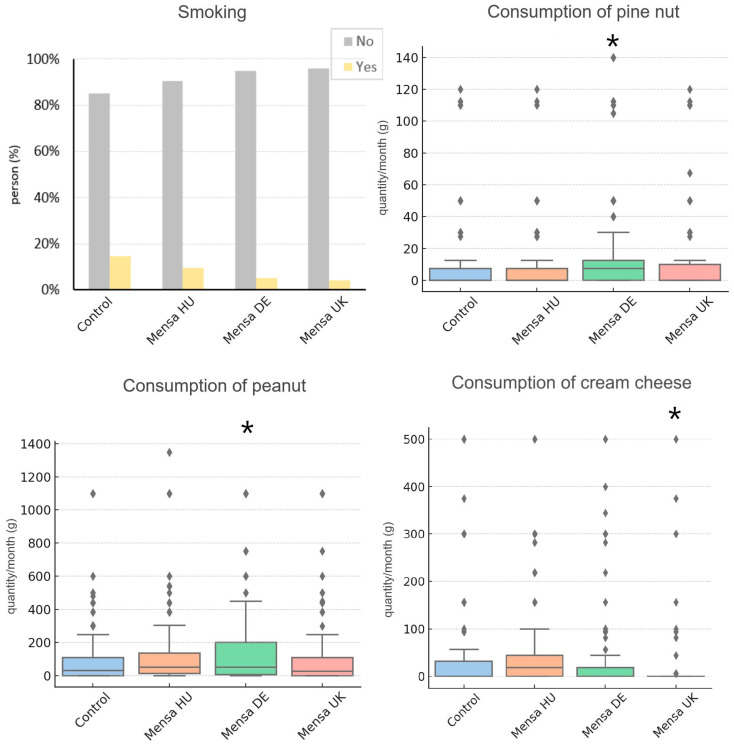
Partial across-country replications among Mensa members in Hungary (HU), Germany (DE), and United Kingdom (UK). Asterisks indicate statistically significant differences (*p* < 0.05) compared to the control group. Diamonds indicate outliers.

We considered a nutrition trend to partially replicate across countries if the direction of effects was concordant and reached corrected statistical significance in at least one country and uncorrected significance in at least another. Smoking was less common among Mensa members in all three groups (OR = 0.29–0.64), an effect that remained significant after correction for multiple testing in the German (*p* = 0.0037) but not the British subsamples (*p* = 0.029). In the Hungarian sample, the effect size was concordant but not statistically significant (*p* = 0.21). For monthly intake of pine nuts, we also found at least trends for higher intake among Mensa members of all countries (B_DE_ = 11.08 g/month, B_UK_ = 7.16 g/month, B_HU_ = 2.10 g/month), a result significant after correcting for multiple testing in the German (*p* = 1.76 × 10^−5^), but not the British subsample (*p* = 0.03), with only a trend among Hungarians (*p* = 0.42). Peanuts were more frequently consumed in the German Mensa subsample (B = 68.02 g/month; *p* = 0.002) and were also more frequently consumed by Hungarian Mensa members (B = 55.28 g/month), but the result was not significant after correction for multiple testing (*p* = 0.016). We observed lower cream cheese intake across all three countries (B_HU_ = −0.47 g/month, B_DE_ = −12.33 g/month, B_UK_ = −34.29 g/month), which was significant only in the British subsample (*p* = 0.009). These findings are shown in Figure 3.

### 3.4. Country-Specific Findings

Country-specific findings were effects that remained significant after correction for multiple testing in a single country. The most country-specific results for food intake were observed among German Mensa members. In this subsample, food diary keeping (OR = 0.35; *p* = 0.013) and dairy-free food consumption (OR = 0.15; *p* = 0.0001) were less frequent. Flaxseed (B = 34.14 g/month; *p* = 0.002) and sunflower (B = 39.15 g/month; *p* = 0.011) were consumed more frequently, while low-fat (<2%) milk was consumed less frequently (B = −7.70 g/month; *p* = 0.0005). Lower meat consumption was observed exclusively in the German Mensa subsample (B = −851.44 g/month), which remained significant after correction (*p* = 9.7 × 10⁻⁵).

Fish (B = 273.06 g/month; *p* = 6.4 × 10^−5^) and seafood (B = 218.99 g/month; *p* = 6.4 × 10^−7^) were more frequently consumed by British Mensa members, without similar significant effects in the other subsamples. Significantly lower intake of poppy seeds (B = −16.98 g/month; *p* = 0.002) and sour cream (including low-fat and fatty products) (B_low-fat_ = −34.62 g/month, *p* = 1.2 × 10^−5^; B_fatty_ = −27.41 g/month, *p* = 0.006) was also observed in the British Mensa subsample without corresponding effects in the others. The consumption of coffee with sugar was significantly lower in the British subsample (OR = 0.27), which remained significant after multiple corrections (*p* = 0.012).

Hungarian Mensa members had a higher monthly intake of goose liver (B = 14.26 g/month) and chicken liver (B = 38.24 g/month) and a higher intake of fatty sour cream (>15%) (B = 47.97 g/month) and cottage cheese (B = 109.39 g/month).

**Figure 4:**
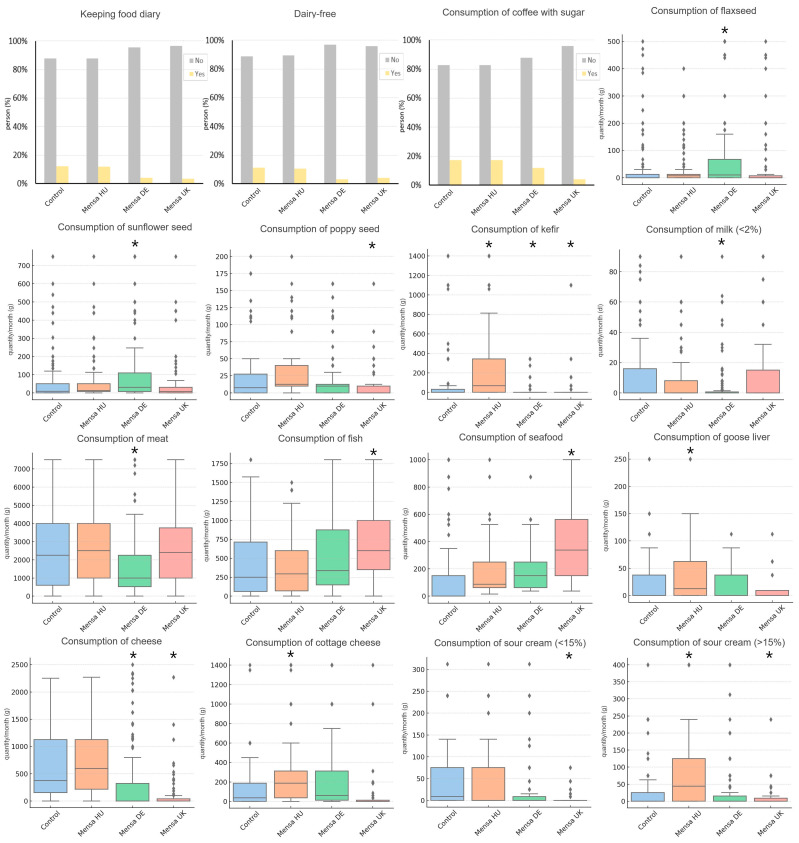
Country-specific findings among Mensa members in Hungary (HU), Germany (DE), and United Kingdom (UK). Asterisks indicate statistically significant differences (*p* < 0.05) compared to the control group. (The figure does not include information on chicken liver consumption due to design considerations.) Diamonds indicate outliers.

Kefir was a unique food item in the sense that its consumption was significantly different between the control and Mensa groups in all three countries, but the direction of effects was not concordant. We recorded higher intakes in Hungary (B = 76.13 g/month; *p* = 0.001), but lower intakes in Germany (B_DE_ = −119.92 g/month; p_DE_ = 4.5 × 10^−7^) and the UK (B_UK_ = −136.57 g/month; p_UK_ = 1.4 × 10^−5^). In the German (B = −354.9 g/month) and British (B = −509.7 g/month) subsamples, lower cheese consumption was observed, and these results remained significant after multiple corrections (p_DE_ = 8.47 × 10⁻^10^; p_UK_ = 3.1 × 10⁻^11^). Country-specific findings are shown in Figure 4.

### 3.5. Reason for Special Diets

Group differences were reported in special (gluten-free, lactose-free, dairy-free, or sugar-free) diets, which can be due to personal or medical reasons. In order to investigate this, in an additional analysis, we compared the frequency of special diets for “personal,” “medical” (pooled from the “diagnosis” and “medical advice” response categories), or “unknown” (no reason listed) between Mensa and control participants. The reason for any special diet was only available as a single variable rather than four separate variables for each of the four special diets. Due to the relatively low number of special diets with indicated reasons (*N* = 71 “medical” and *N* = 87 “personal,” *N* = 78 “unknown” reasons), we did not perform analyses separately per country. In three logistic regression models, we calculated the odds of following a special diet either for a medical, personal, or unknown reason as a function of group membership, controlling for age and sex. Control participants were significantly more likely to report a special diet due to medical reasons (OR = 3.79; *p* = 10^−5^), but less likely to report it for personal reasons (OR = 0.35; *p* = 0.004), with no significant effect for unknown reasons (OR = 0.7; *p* = 0.265). In sum, we found that special diets are more common among Mensa members for personal, but less common for medical reasons.

### 3.6. Alternative Analyses

#### 3.6.1. Different Outlier Handling

To ensure the robustness of our findings, we conducted alternative analyses with different methods for handling extreme values. In our original analyses, we excluded nutritional values at least 2 SD above the mean to eliminate implausible entries likely resulting from error. In alternative analyses, we repeated analyses using a 3 SD cutoff instead (Supplementary Table S5). We also calculated results using no such outlier filtering (Supplementary Table S6). These alternative approaches revealed similar patterns as the main analyses, confirming that the reported trends are not artifacts of the outlier-handling method. However, in the absence of any outlier detection, certain extreme values (e.g., unusually high monthly intakes) influenced the results disproportionately, underscoring the importance of robust data cleaning procedures. These findings further validate the reliability of the observed trends across countries.

#### 3.6.2. Controlling for Years of Education

To further test the robustness of our results, we conducted an additional analysis to assess whether the association between Mensa membership and dietary habits could be attributed to differences in educational attainment. Participants’ education levels were originally reported as categorical variables and were converted to estimated years of education (10, 12, 15, 17, or 21 years, depending on the highest attained degree). We repeated the main regression analyses, including years of education, as an additional covariate alongside age and sex. The inclusion of educational level did not meaningfully alter the pattern or significance of the reported effects (Supplementary Table S7). This suggests that the observed dietary differences between Mensa members and control participants are not primarily driven by differences in educational attainment.

#### 3.6.3. Hungarian-Only Sample

As our monoethnic Mensa samples were compared to an international control sample, country differences in nutritional habits can confound the differences we found between controls and Mensa members. As true Mensa membership or intelligence effects are likely to replicate across countries, confounding effects are not. Our primary analytical strategy was to compare Mensa members from three different countries to the international control sample and focus on effects that replicate across multiple Mensa groups. However, as the majority (58.16%) of control participants were recruited from Hungary, a country with an available Mensa group, in an alternative analysis, we sought to replicate international findings in a monoethnic analytical sample comprised of only Hungarian controls and Mensa participants. This design eliminates country confounding at the cost of reduced statistical power due to the smaller control sample. Of the 9 findings that we considered internationally replicated (see Section 3), 4 were significant (*p* < 0.05) in the original comparison of Hungarians to international controls, 3 of which replicated in the monoethnic re-analysis. The sign of effects was consistent with international trends and original analyses in 8 out of 9 cases (Supplementary Table S8). As results largely replicate in a monoethnic sample, it is likely that most country confounding was successfully eliminated in the original analyses by focusing on trends that replicate internationally.

#### 3.6.4. Cross-Country Robustness Analyses

To further assess the robustness and generalizability of Mensa-related dietary patterns, we conducted two complementary analyses: (1) national Mensa subgroups (Hungary, Germany, and United Kingdom) were compared to the full international control group, and (2) the same comparisons were repeated using only Hungarian control participants to reduce cultural variability.

Results (see Supplementary Table S9) indicate that certain associations—such as reduced consumption of plant-based drinks—were consistent across all Mensa subgroups and control types, suggesting robust group-level differences. For other variables, such as sugar-free product use and yoghurt consumption, results were directionally similar but varied in effect size or significance depending on the control group. Additional variables (e.g., egg consumption) appeared to be sensitive to the choice of comparison group, as they emerged only in one of the two analyses.

These findings support the partial cross-national validity of several dietary differences while highlighting the importance of control group selection when assessing culturally influenced habits.

## 4. Discussion

Numerous studies have demonstrated the association between higher intelligence and improved health outcomes, including lower mortality rates (Fries and Pietschnig 2022; Christensen et al. 2016; Batty et al. 2007a). However, the underlying mechanisms driving this relationship remain unclear, and various potential explanations have been proposed. One hypothesis posits that macro- and micro-environmental factors, along with personal characteristics, foster healthier lifestyle choices, thereby contributing to better health (McKinlay 2023). Another perspective suggests that health literacy is a critical determinant of lifestyle, with individuals possessing higher cognitive abilities—such as those with higher IQs—being better equipped to comprehend health-related information and make informed, health-promoting decisions (Gottfredson 1998; Neisser et al. 1996). A further viewpoint contends that superior living conditions in adulthood, often associated with higher IQ, provide the foundation for healthier life activities (Herrnstein and Murray 1994; Strenze 2007). These theories, although different in their specific proposed mechanism, collectively attribute a significant influence on health behaviours, which, in turn, can lead to improved health outcomes. Under this hypothesis, high-IQ individuals should have better health behaviours, and this is the proximal reason for better health outcomes they eventually experience. An alternative explanation centers on the genetic and biological underpinnings of individuals with higher IQs (Deary 2012). For example, certain biomarkers (e.g., blood glucose and blood pressure) have been shown to correlate with intelligence, suggesting that higher cognitive ability may be linked to better health outcomes due to more favorable physiological profiles (Schaefer et al. 2016; Sörberg et al. 2014). Under this hypothesis, conversely, health behaviour is not necessarily better in high-IQ individuals: better health outcomes are caused by the biological causes of intelligence, not the lifestyle choices intelligence fosters or enables.

In the present study, lifestyle habits, frequency of consumption, and amount of several staples were measured in individuals with IQ 130 or above in Hungary, Germany, and the United Kingdom and compared with an international control group. Additional analyses suggest that educational attainment does not substantially account for the relationship between Mensa membership and dietary habits, as the majority of associations remained significant even after controlling for education.

One of the key findings of our study is that Mensa members have lower smoking rates. This is in line with the results of other studies that have found a negative correlation between smoking behaviour and intelligence (Hemmingsson et al. 2008; Kubicka et al. 2001; Wennerstad et al. 2010). Our results may reflect a pattern often interpreted as more rational decision-making among individuals with higher cognitive ability, such as avoiding tobacco use. However, as we did not directly assess decision-making processes, this interpretation should be treated with caution.

In all three countries, consumption of special foods was lower among Mensa members, a trend that held across all three countries. Of the four diets we surveyed—gluten-free, sugar-free, lactose-free, and dairy-free—all were significantly less common in the German subsample and gluten-free, sugar-free, and lactose-free in the English subsample, while gluten-free was significant in the Hungarian subsample. Additional analyses showed that Mensa members primarily follow special diets due to personal, not medical, reasons. Special diets due to medical reasons were, in fact, more common in the control sample. It is important to consider the role of fad diets and illusory health literacy in the context of dietary choices. As mentioned in one of the theories above, the theory of health literacy and optimal living conditions is nuanced by the fact that nutrition is a constantly evolving field, and the quality of information about food in public discourse is variable. Navigating through information can be particularly challenging for individuals without professional knowledge. Even if someone possesses high intelligence or health consciousness, they may still fail to adhere to evidence-based guidelines established by scientific consensus. Without proper information, they may invest time, energy, and money in unsupported practices, such as fad diets, that they have encountered on the internet or other media, which do not contribute to health promotion. Moreover, our study relied on self-reported, declarative data, which may differ from actual dietary behavior. Therefore, no firm conclusions can be drawn about the real-life implementation or consistency of the reported habits. This phenomenon underscores the importance of health literacy, which not only involves understanding nutrition-related information but also the critical thinking necessary to identify evidence-based recommendations and avoid misleading health claims.

Higher oilseed intakes were measured among Mensa members in all three countries. Regular consumption of these staples can have beneficial physiological effects at least in some studies (Akl 2023). The dietary popularity of oilseeds and dry pulses is helped by the reduced intake recommendations for meat and meat products (Bonnet and Coinon 2024) and the United Nations (UN) recommendation to balance animal and vegetable protein intakes (World Health Organization 2024a). Consumers and the industry are also increasingly using various seeds and dried pulses to produce meat-free or meat-reduced meals and food products (Ahmad et al. 2022; Andreani et al. 2023). The data on meat and oilseed consumption observed in the German Mensa subsample suggest that sustainability considerations may play a crucial role in dietary decisions, as these individuals appear to reduce meat consumption and replace it with oilseeds. In contrast, no similar trend was observed in the Hungarian and British samples, where the increased consumption of oilseeds was associated with higher intakes of meat and meat products. Our findings further support the notion that the consumer-level interpretation of nutrition-related information is often not grounded in solid scientific knowledge. Nutritional guidelines promoted in the media and on the internet, often lacking scientific evidence, strongly influence individuals’ decisions, leading them to not necessarily follow evidence-based recommendations established by scientific consensus. This trend is further reinforced by the fact that dietary choices among Hungarian and British Mensa members appear to reflect social status-driven trends rather than scientifically grounded decisions aimed at improving health. Thus, our results support the theory that even highly intelligent or health-conscious individuals often struggle to effectively navigate the vast array of nutrition-related information available.

Mensa subsamples from all three countries had lower egg intakes. Even though eggs are part of the cuisine of all three nations, are a readily available staple, and are also a traditional dish (e.g., English breakfast), there was still a significant negative difference in consumption. Daily intake was half as high for Mensa members as for control group participants (16.84%). One possibility is that Mensa members prefer less conventional breakfast food items, resulting in relatively lower intake of eggs.

Our work has identified several country-specific dietary habits related to an increased consumption of country-specific stables in two out of the three countries. Hungarian Mensa members had a higher intake of goose liver, fatty sour cream, and poppy seeds, which are mainstays of Hungarian cuisine. In a similar way, British individuals had significantly higher intakes of fish and seafood, which are similar stables of British cuisine. These findings may suggest a preference of typical local foods and a decreased preference for international fad diets. While here is little evidence in the literature about a link between high IQ and tradition-keeping, people with high IQ may still consider local food staples a more authentic and preferable dietary choice.

In general, the findings of our study provide little evidence that Mensa members have broadly better health behaviour. For example, we found no difference compared to control participants in physical activity, body mass index, and consumption of fruit and vegetables. While many robust differences were found in Mensa members, which replicated across countries, these appear informed more by fashion than health, such as a preference for special diets and local staples. These findings are in line with previous studies (Fries and Pietschnig 2022; Arden et al. 2016; Hagenaars et al. 2016; Bratsberg and Rogeberg 2017), which found that intelligence is less robustly associated with better health behaviour than with better health itself. Of the two competing explanations for better health in the highly intelligent (direct effects of intelligence or common biological causes), this observation is more in line with the latter and suggests that lower morbidity and mortality in the highly intelligent is not a direct consequence of lifestyle choices high intelligence enables. This is because better health behavior in highly intelligent individuals would support the direct effects hypothesis, implying that higher intelligence leads to better health through health-preserving behaviors. Conversely, the opposite pattern, where higher-IQ individuals do not exhibit better health behavior, requires an indirect explanation (i.e., common causes hypothesis), suggesting that better health outcomes may result from shared biological or genetic factors rather than from lifestyle choices. Instead, high intelligence and low morbidity/mortality may both result from a broadly healthier physical constitution of the individual (Deary et al. 2021). It is important to note the limitations of our work. The Mensa community, while composed of individuals with high IQs, is not representative of all individuals with elevated cognitive abilities, and this may introduce potential biases into our results. Because the control group was highly international, we cannot exclude that differences in eating habits compared to one monoethnic Mensa group are due to country-related, not intelligence-related, differences in eating habits. This is, however, very unlikely if the difference replicates in another or both other monoethnic Mensa groups from further countries. While some observed associations appeared sensitive to the composition of the control group, several key dietary trends (e.g., reduced consumption of plant-based drinks and sugar-free products among Mensa members) remained significant or directionally consistent when restricting analyses to a monoethnic Hungarian sample. This supports the interpretation that the main effects are not solely driven by cultural or national differences, although the potential influence of control group composition on certain comparisons warrants careful interpretation. Supporting this notion, research by Karpinski et al. (Karpinski et al. 2018) on Mensa members in the United States also found poorer health outcomes among those with higher intelligence. However, in a broader, non-selected sample, they observed that individuals with higher IQs generally reported better health outcomes (Williams et al. 2023). The limited sample size and reliance on self-report measures present significant methodological challenges in this study. Consequently, it remains unclear whether the observed results are meaningfully associated with intelligence or membership in Mensa. Further research with a larger, more diverse sample and objective assessments is needed to clarify these relationships.

## 5. Conclusions

This study explores the relationship between high intelligence and dietary habits among Mensa members in Hungary, Germany, and the United Kingdom. It finds that Mensa members tend to have lower smoking rates and consume fewer special diets (e.g., gluten-free or sugar-free) compared to the control group, suggesting that cognitive ability may influence health-conscious decisions. The study also reveals higher intakes of oilseeds and a lower consumption of eggs among Mensa members, with country-specific dietary patterns observed. However, despite their higher cognitive abilities, Mensa members do not exhibit significantly healthier overall dietary behaviours, which may indicate that cognitive ability does not directly correlate with healthier lifestyle choices. The study’s limitations, including sample size and self-reporting, call for further research to better understand these associations.

## Data Availability

The original data presented in the study are openly available in Mendeley Data at https://doi.org/10.17632/tn35cg3y3h.1.

## Supplementary Materials

The following supporting information can be downloaded at: https://www.mdpi.com/article/10.3390/jintelligence13060067/s1, Supplementary Table S1: Self-report variable questions and their response options before the dietary habits section; Supplementary Table S2: Response options of frequency of consumption and amount consumed for each product; Supplementary Table S3: Frequency of consumption; Supplementary Table S4: Detailed statistics for each variable; Supplementary Table S5: Detailed statistics with the 3SD cutoff; Supplementary Table S6: Detailed statistics without outlier filtering; Supplementary Table S7: Comparison of effect sizes and *p*-values before and after controlling for years of education for across-country replicated variables; Supplementary Table S8: Comparison of effect sizes and *p*-values in the original and Hungarian-only analyses for across-country replicated variables; Supplementary Table S9: Comparison of effect sizes and *p*-values in national Mensa vs. control group analyses using international and Hungarian-only controls.
