# Supplementary material for: Intelligence and Dietary Habits: An International Study of Mensa Members
Source: J Intell. 2025 Jun 10;13(6):67. doi: 10.3390/jintelligence13060067 (PMC12194576; doi:10.3390/jintelligence13060067)
Supplement: Supplementary file 1 [file jintelligence-13-00067-s001.zip › jintelligence-3475719-supplementary.pdf]

Supplementary Table S1: Self-report variable questions and their response options before the dietary habits section

Question	Response options	Comment
Age (years)	[free-form continuous data entry]	
Gender	„male”,<br>„female”,<br>„other”	
Weight	[free-form continuous data entry]	These data were provided in the units of measurement officially used in the region. The data converted into metric and the body mass index (BMI) was determined.
Height	[free-form continuous data entry]	
Place of residence	"capital",<br>"agglomeration",<br>"city",<br>"town",<br>"village"	
Highest degree	"under High School", "High School", "College/University", Master's degree", "PhD"	
Do you smoke?	"yes",<br>"no"	If the answer is "yes", the next question was: How long have you been smoking? (year)
Diagnosed diseases:	[free-form continuous data entry]	This was an open question, so respondents could enter the diseases they had been diagnosed with.
Have you had surgery affecting the digestive system (e.g. stomach, gut, pancreas, bile)?	"yes",<br>"no"	
How much time do you spend exercising, playing sports, training each week? (hours/week)	„less than 1 hour”, „1”, „1,5”, „2”, ”2,5, „3”, „3,5”, „4”, „4,5”, „5”, „5,5”, „6”, „6,5”, „7”, „7,5”, „8”, „8,5”, „9”, „9,5”, „10”, „10,5”, „11”, „11,5”, „12”, „12,5”, „13”, „13,5”, „14”, „14,5”, „15”, „more than 15 hours”	
Apart from sports, how often do you do physical activity, such as gardening, housework, when your heart beats faster, you get hot, you sweat for at least 10 minutes?	"several times a week", "three times a week",<br>"twice a week",<br>"once a week",<br>"less than once a week"	
Do you monitor your daily movements using a smart device? (e.g. watch, phone, etc.)	"yes",<br>"no"	
How many steps do you take on average per day?	"<5000";<br>"5000-7499",<br>"7500-9999",<br>"10000-12499",<br>">12500",<br>"I don't know"	

Supplementary Table S2: Response options of frequency of consumption and amount consumed for each product

Food	Frequency of consumption response options
lentil (all types)	„many times a day”<br>„once a day”<br>„more than 6 times a week but not daily”<br>„4-6 times a week”<br>„1-3 times a week”<br>„6-8 times a month but not weekly”<br>„4-5 times a month but not weekly”<br>„1-3 times a month”<br>„less often”<br>„never”
dried beans	
yellow peas	
chickpeas	
soy	
"white" baked goods (bread/bread roll/baguette)	
whole grain baked goods (bread/bread roll/baguette)	
pastry	
puffed cereal products	
"white" pasta	
whole grain pasta	
bulgur	
millet	
couscous	
white rice	
brown rice	
corn	
quinoa	
buckwheat	
muesli	
bran	
green vegetables	
dairy desserts	
plant-based cream	

Food	Frequency of consumption response options	Amount consumed per dish response options
walnut	„many times a day”<br>„once a day”<br>„more than 6 times a week but not daily”<br>„4-6 times a week”<br>„1-3 times a week”<br>„6-8 times a month but not weekly”<br>„4-5 times a month but not weekly”<br>„1-3 times a month”<br>„less often”<br>„never”	„a few”<br>„little handful”<br>„big handful”<br>„little bag”<br>„big bag”<br>„only seeded baked goods”
pecan		
almond		
sesame seed		
linseed		
pumpkin seed		
pine nut		
peanut		
sunflower seed		
pistachio		
chia seed		
poppy seed		
chicken egg	„many times a day”<br>„once a day”<br>„more than 6 times a week but not daily”<br>„4-6 times a week”<br>„1-3 times a week”<br>„6-8 times a month but not weekly”<br>„4-5 times a month but not weekly”<br>„1-3 times a month”<br>„less often”<br>„never”	„1”, „2”, „3”, „4”, „5”, „6”, „7”, „8”, „9”, „10”, „more than 10”

Food	Frequency of consumption response options	Amount consumed per dish response options
meat products<br>chitterlings<br>chicken liver<br>goose liver<br>meat<br>fish<br>seafood	„many times a day”<br>„once a day”<br>„more than 6 times a week but not daily”<br>„4-6 times a week”<br>„1-3 times a week”<br>„6-8 times a month but not weekly”<br>„4-5 times a month but not weekly”<br>„1-3 times a month”<br>„less often”<br>„never”	„less than 50 g”<br>„50-100 g”<br>„101-150 g”<br>„151-200 g”<br>„more than 200 g”
fruits<br>vegetables	„many times a day”<br>„once a day”<br>„more than 6 times a week but not daily”<br>„4-6 times a week”<br>„1-3 times a week”<br>„6-8 times a month but not weekly”<br>„4-5 times a month but not weekly”<br>„1-3 times a month”<br>„less often”<br>„never”	„less than 100 g”<br>„100-250 g”<br>„251-500 g”<br>„more than 500 g”
skimmed/semi-skimmed milk (<2%)<br>whole milk (>2%)	„many times a day”<br>„once a day”<br>„more than 6 times a week but not daily”<br>„4-6 times a week”<br>„1-3 times a week”<br>„1-5 times a month but not weekly”<br>„less often”<br>„never”	„less than 0,5 dl”; „0,5 dl”;„1 dl”; „1,5 dl”; „2 dl”; „2,5 dl”; „3 dl”; „3,5 dl”; „4 dl”; „4,5 dl”; „5 dl”; „5,5 dl”; „6 dl”; „6,5 dl”; „7 dl”; „7,5 dl”; „8 dl”; „8,5 dl”; „9 dl”; „9,5 dl”; „10 dl”; „more than 10 dl”
natural yoghurt<br>flavoured (e.g. fruit) yoghurt/yoghurt drink	„many times a day”<br>„once a day”<br>„more than 6 times a week but not daily”<br>„4-6 times a week”<br>„1-3 times a week”<br>„1-5 times a month but not weekly”<br>„less often”<br>„never”	„less than 125 g (e.g. a small cup of joghurt)”<br>„125-150 g”<br>„151-300 g”<br>„301-350 g”<br>„351-500 g (e.g.a big cup of yoghurt)<br>„more than 500 g (e.g. a bucket/pot of yoghurt)”
kefir		„less than 125 g”<br>„125-150 g”<br>„151-300 g”<br>„301-350 g”<br>„351-500 g”<br>„more than 500g”
reduced fat soured cream (<15%)<br>soured cream (>15%)	„many times a day”<br>„once a day”<br>„more than 6 times a week but not daily”<br>„4-6 times a week”<br>„1-3 times a week”<br>„1-5 times a month but not weekly”<br>„less often”<br>„never”	„15-20 g (approx one tablespoon)”<br>„21-40 g (approx two tablespoon)”<br>„41-60 g”<br>„61-100 g”<br>„more than 100 g”
cream	„many times a day”<br>„once a day”<br>„more than 6 times a week but not daily”<br>„4-6 times a week”<br>„1-3 times a week”<br>„1-5 times a month but not weekly”<br>„less often”<br>„never”	„less than 0,5 dl”<br>„0,51-1 dl”<br>1,1-1,5 dl”<br>„1,51-2 dl”<br>„2,1-2,5 dl”<br>„more than 2,5 dl”
cottage cheese	„many times a day”<br>„once a day”<br>„more than 6 times a week but not daily”<br>„4-6 times a week”<br>„1-3 times a week”<br>„1-5 times a month but not weekly”<br>„less often”<br>„never”	„1-50 g (approx two tablespoons)”<br>„51-100 g (approx 3-4 tablespoons)”<br>„101-150 g”<br>„more than 150 g”

Food	Frequency of consumption response options	Amount consumed per dish response options
cheese<br>cheese cream	„many times a day”<br>„once a day”<br>„more than 6 times a week but not daily”<br>„4-6 times a week”<br>„1-3 times a week”<br>„1-5 times a month but not weekly”<br>„less often”<br>„never”	„1-25 g (e.g. a medium slice)”<br>„26-50 g (e.g. two medium slices)”<br>„51-75 g”<br>„76-100 g”<br>„101 -125 g”<br>„126-150 g”<br>„more than 150 g”
plant-based drink	„many times a day”<br>„once a day”<br>„more than 6 times a week but not daily”<br>„4-6 times a week”<br>„1-3 times a week”<br>„1-5 times a month but not weekly”<br>„less often”<br>„never”	„less than 0,5 dl”; „0,5 dl”; „1 dl”; „1,5 dl”; „2 dl”; „2,5 dl”; „3 dl”; „3,5 dl”; „4 dl”; „4,5 dl”; „5 dl”; „5,5 dl”; „6 dl”; „6,5 dl”; „7 dl”; „7,5 dl”; „8 dl”; „8,5 dl”; „9 dl”; „9,5 dl”; „10 dl”; „more than 10 dl”
plant-based “yoghurt”	„many times a day”<br>„once a day”<br>„more than 6 times a week but not daily”<br>„4-6 times a week”<br>„1-3 times a week”<br>„1-5 times a month but not weekly”<br>„less often”<br>„never”	„less than 125 g”<br>„125-150 g”<br>„151-300 g”<br>„301-350 g”<br>„351-500 g”<br>„more than 500 g”
coffee		„1 cup per day”<br>„2 cups per day”<br>„3 cups per day”<br>„4 cups per day”<br>„5 cups per day”<br>„more than 5 cups per day”<br>„I do not consume”

Supplementary Table S3: Frequency of consumption

Variable	Total			Control			Mensa Hungary			Mensa Germany			Mensa United Kingdom		
	N	Mean	SD	N	Mean	SD	N	Mean	SD	N	Mean	SD	N	Mean	SD
Weight (kg)	745	76,36	15,57	196	69,58	15,32	190	75,64	15,64	257	76,84	15,75	142	75,08	14,14
Height (cm)	761	172,64	8,81	196	169,58	9,10	190	171,75	8,25	257	174,22	8,05	142	171,88	9,52
BMI (kg/m^2)	753	25,31	4,31	196	23,86	4,36	190	25,51	4,24	257	25,24	4,30	142	25,39	4,14
Smoke	785	0,08		196	0,15		190	0,09		257	0,05		142	0,04	
Food diary	785	0,08		196	0,12		190	0,12		257	0,04		142	0,04	
Special food	783	0,30		196	0,35		190	0,33		257	0,27		142	0,24	
Gluten-free	707	0,07		196	0,21		190	0,08		257	0,03		142	0,04	
Lactose-free	710	0,14		196	0,26		190	0,20		257	0,08		142	0,04	
Dairy-free	709	0,08		196	0,18		190	0,11		257	0,03		142	0,04	
Sugar-free	704	0,08		196	0,23		190	0,12		257	0,03		142	0,01	
Walnut (g/month)	749	118,01	176,38	196	108,04	199,38	190	82,43	136,19	257	125,29	197,16	142	62,20	146,33
Pecans (g/month)	740	25,30	45,91	196	17,69	42,61	190	14,99	37,20	257	22,82	51,31	142	23,61	49,48
Almond (g/month)	754	136,20	252,83	196	130,42	259,86	190	89,59	162,47	257	135,91	264,57	142	136,36	312,75
Sesame seed (g/month)	727	46,62	59,06	196	44,11	58,81	190	41,32	59,54	257	44,35	54,90	142	35,60	65,60
Flaxseed (g/month)	723	76,34	100,68	196	34,14	87,23	190	31,60	65,41	257	69,73	129,07	142	33,03	94,58
Pumpkin seed (g/month)	752	88,76	199,53	196	70,46	204,97	190	91,97	217,19	257	90,10	184,88	142	67,66	195,02
Pine nut (g/month)	732	15,22	23,25	196	8,14	22,92	190	7,95	17,35	257	16,75	26,36	142	11,15	23,38
Peanuts (g/month)	747	139,61	204,25	196	92,25	164,10	190	136,00	222,61	257	143,26	212,19	142	109,79	204,95
Sunflower seed (g/month)	746	80,36	140,58	196	67,70	134,83	190	64,49	120,45	257	95,50	164,94	142	45,84	117,91
Pistachio (g/month)	752	58,98	115,75	196	77,80	145,16	190	67,88	119,69	257	40,26	91,13	142	43,75	105,59
Chia seed (g/month)	742	42,54	78,24	196	51,85	103,94	190	26,10	67,94	257	24,83	65,25	142	19,95	72,09
Poppy seed (g/month)	734	25,90	38,57	196	26,00	48,35	190	31,57	42,46	257	19,36	32,77	142	10,97	24,62
Oil seeds (g/month)	727	951,48	981,47	196	786,88	910,42	190	870,19	958,97	257	1043,20	1084,03	142	711,44	853,29
Egg (piece/month)	690	19,69	17,29	196	24,93	19,35	190	21,65	18,22	257	13,64	13,24	142	17,27	16,37
Meat products (g/month)	634	1323,12	1331,07	196	1206,82	1355,25	190	1639,31	1418,97	257	987,34	1153,34	142	1374,18	1333,41
Meat (g/month)	673	2593,42	2039,73	196	2469,26	2246,80	190	2848,67	2035,59	257	1463,46	1604,84	142	2586,99	1983,62
Fish (g/month)	682	554,97	445,18	196	439,31	438,83	190	427,80	397,27	257	504,66	441,01	142	738,00	464,18
Sea food (g/month)	453	224,98	251,48	196	152,24	253,20	190	213,94	228,78	257	203,11	180,98	142	382,40	284,47
Chicken liver (g/month)	440	87,70	118,99	196	72,50	114,59	190	112,29	134,63	257	39,17	71,24	142	65,13	126,90
Goose liver (g/month)	450	30,60	40,93	196	22,19	43,09	190	35,76	43,32	257	20,12	31,80	142	15,63	31,12
Milk <2% (dl/month)	730	14,63	20,74	196	13,07	23,68	190	10,67	21,01	257	4,36	12,77	142	13,85	25,80
Milk >2% (dl/month)	730	10,62	18,64	196	9,15	18,98	190	11,14	20,64	257	7,71	18,74	142	3,42	13,83
Sour cream <15% (g/month)	760	30,53	58,06	196	39,30	62,23	190	49,34	78,99	257	15,32	43,61	142	4,26	12,99
Sour cream >15% (g/month)	755	38,66	74,74	196	31,56	68,50	190	81,04	106,14	257	25,58	55,88	142	7,59	24,12
Cream (g/month)	651	1,50	2,52	196	1,12	2,01	190	1,16	2,13	257	1,83	3,02	142	1,74	2,92
Cottage cheese (g/month)	755	156,18	268,96	196	117,33	200,45	190	216,49	283,36	257	212,87	325,35	142	32,54	148,94
Cheese (g/month)	755	463,96	588,08	196	646,66	633,96	190	711,26	598,25	257	271,39	522,69	142	94,41	269,48
Cream cheese (g/month)	745	49,91	92,98	196	52,53	112,50	190	50,27	89,34	257	37,12	92,22	142	12,77	61,08
Natural yoghurt (g/month)	702	602,19	645,83	196	552,81	812,87	190	374,64	632,39	257	247,22	519,47	142	259,25	533,18
Flavoured yoghurt (g/month)	734	201,16	346,96	196	182,39	342,57	190	213,86	363,83	257	117,33	281,95	142	155,50	423,29

Variable	Total			Control			Mensa Hungary			Mensa Germany			Mensa United Kingdom		
	N	Mean	SD	N	Mean	SD	N	Mean	SD	N	Mean	SD	N	Mean	SD
Kefir (g/month)	765	87,39	234,39	196	94,58	274,23	190	201,11	332,53	257	12,50	46,90	142	27,92	139,60
Plant-based drink (dl/month)	658	12,92	25,51	196	28,57	31,39	190	9,53	23,11	257	10,25	25,36	142	7,30	20,33
Fruits (g/month)	679	6825,89	4390,65	196	5725,44	4372,09	190	5436,98	4237,17	257	5704,44	4301,70	142	6153,10	4823,33
Vegetable (g/month)	764	8163,55	6604,70	196	8362,43	7309,38	190	6919,12	6030,98	257	8298,61	6301,47	142	9326,26	6683,45
Coffee (cup/day)	768	3,75	2,40	196	3,46	2,50	190	3,54	2,35	257	4,03	2,35	142	3,95	2,37
Coffee (black)	783	0,23		196	0,19		190	0,13		257	0,28		142	0,32	
Coffee with milk	785	0,41		196	0,40		190	0,47		257	0,37		142	0,40	
Coffee with plant-based drink	785	0,14		196	0,20		190	0,16		257	0,13		142	0,06	
Coffee with sugar	785	0,13		196	0,17		190	0,17		257	0,12		142	0,04	
Coffee with sweetener	785	0,09		196	0,12		190	0,17		257	0,03		142	0,05	
Coffee with honey	785	0,03		196	0,03		190	0,08		257	0,00		142	0,02	

Supplementary Table S4: Detailed statistics for each variable

Variable	Mensa Hungary				Mensa Germany				Mensa United Kingdom				Effect size type
	Effect size	95% CI-l	95% CI-u	p	Effect size	95% CI-l	95% CI-u	p	Effect size	95% CI-l	95% CI-u	p	
Weight (kg)	1,31	-1,70	4,33	0,39	2,61	-0,30	5,53	0,08	-3,06	-6,98	0,86	0,13	Mean difference
Height (cm)	0,38	-1,13	1,89	0,62	3,32	1,88	4,77	0,00	0,02	-1,92	1,96	0,98	Mean difference
BMI (kg/m2)	0,43	-0,47	1,33	0,35	0,09	-0,79	0,96	0,85	-0,90	-2,06	0,26	0,13	Mean difference
Smoke	0,64	0,32	1,28	0,21	0,32	0,15	0,69	0,00	0,29	0,10	0,89	0,03	Odds ratio
Food diary	1,12	0,56	2,20	0,75	0,36	0,16	0,81	0,01	0,31	0,10	1,02	0,05	Odds ratio
Special food	1,11	0,70	1,76	0,67	0,80	0,51	1,26	0,34	0,78	0,42	1,44	0,43	Odds ratio
Gluten-free	0,33	0,15	0,71	0,00	0,11	0,04	0,27	0,00	0,13	0,04	0,45	0,00	Odds ratio
Lactose-free	0,98	0,54	1,78	0,95	0,35	0,18	0,69	0,00	0,19	0,06	0,57	0,00	Odds ratio
Dairy-free	0,74	0,36	1,52	0,41	0,15	0,06	0,40	0,00	0,35	0,11	1,09	0,07	Odds ratio
Sugar-free	0,54	0,27	1,06	0,07	0,10	0,04	0,25	0,00	0,05	0,01	0,27	0,00	Odds ratio
Walnut (g/month)	-24,37	-63,39	14,65	0,22	17,04	-20,57	54,65	0,37	-44,28	-93,64	5,08	0,08	Mean difference
Pecans (g/month)	-2,89	-13,25	7,47	0,59	4,60	-5,42	14,62	0,37	4,78	-8,29	17,84	0,47	Mean difference
Almond (g/month)	-22,32	-78,20	33,56	0,43	21,10	-32,73	74,93	0,44	36,05	-34,44	106,54	0,32	Mean difference
Sesame seed (g/month)	-0,23	-13,56	13,10	0,97	3,30	-9,60	16,20	0,62	-3,90	-20,76	12,95	0,65	Mean difference
Flaxseed (g/month)	-2,74	-24,87	19,38	0,81	34,14	12,50	55,78	0,00	-6,30	-34,55	21,95	0,66	Mean difference
Pumpkin seed (g/month)	21,80	-22,88	66,47	0,34	21,72	-21,01	64,44	0,32	-0,18	-56,29	55,93	1,00	Mean difference
Pine nut (g/month)	2,10	-3,04	7,25	0,42	11,08	6,06	16,10	0,00	7,16	0,64	13,68	0,03	Mean difference
Peanuts (g/month)	55,28	10,18	100,38	0,02	68,02	24,36	111,67	0,00	48,43	-8,68	105,54	0,10	Mean difference
Sunflower seed (g/month)	7,32	-24,05	38,69	0,65	39,15	9,02	69,28	0,01	-3,39	-42,91	36,13	0,87	Mean difference
Pistachio (g/month)	-0,11	-25,84	25,62	0,99	-25,43	-50,22	-0,63	0,04	-11,78	-44,29	20,72	0,48	Mean difference
Chia seed (g/month)	-18,01	-35,27	-0,75	0,04	-19,07	-35,80	-2,34	0,03	-18,04	-39,96	3,87	0,11	Mean difference
Poppy seed (g/month)	4,84	-3,88	13,57	0,28	-7,43	-15,70	0,84	0,08	-16,98	-27,87	-6,09	0,00	Mean difference
Oil seeds (g/month)	161,09	-61,83	384,01	0,16	342,27	126,95	557,59	0,00	78,65	-199,96	357,27	0,58	Mean difference
Egg (piece/month)	-4,31	-8,15	-0,48	0,03	-12,21	-15,87	-8,54	0,00	-9,50	-14,33	-4,67	0,00	Mean difference
Meat products (g/month)	339,78	50,89	628,67	0,02	-324,45	-610,58	-38,33	0,03	7,96	-388,04	403,96	0,97	Mean difference
Meat (g/month)	497,12	62,67	931,58	0,03	-851,45	-1277,22	-425,68	0,00	490,06	-93,36	1073,49	0,10	Mean difference
Fish (g/month)	-28,01	-125,69	69,68	0,57	47,54	-49,27	144,36	0,34	273,06	139,93	406,18	0,00	Mean difference
Sea food (g/month)	58,68	-10,38	127,74	0,10	47,40	-16,38	111,19	0,15	218,98	134,02	303,95	0,00	Mean difference
Chicken liver (g/month)	38,24	9,34	67,15	0,01	-34,32	-67,91	-0,73	0,05	-10,22	-73,88	53,44	0,75	Mean difference
Goose liver (g/month)	14,26	4,35	24,16	0,01	-0,77	-12,34	10,81	0,90	-4,68	-26,32	16,97	0,67	Mean difference
Milk <2% (dl/month)	-1,48	-6,01	3,04	0,52	-7,71	-12,08	-3,33	0,00	2,75	-3,19	8,68	0,36	Mean difference
Milk >2% (dl/month)	2,72	-1,40	6,84	0,20	-0,35	-4,31	3,61	0,86	-3,80	-9,07	1,46	0,16	Mean difference
Sour cream <15% (g/month)	10,60	-1,67	22,88	0,09	-23,69	-35,40	-11,99	0,00	-34,62	-50,06	-19,18	0,00	Mean difference
Sour cream >15% (g/month)	47,97	32,32	63,63	0,00	-7,38	-22,23	7,48	0,33	-27,41	-47,19	-7,64	0,01	Mean difference
Cream (g/month)	0,34	-0,21	0,89	0,22	1,05	0,49	1,61	0,00	1,19	0,37	2,00	0,00	Mean difference
Cottage cheese (g/month)	109,39	52,97	165,81	0,00	106,16	51,44	160,88	0,00	-68,27	-140,37	3,83	0,06	Mean difference
Cheese (g/month)	75,75	-41,61	193,12	0,21	-354,91	-466,82	-243,00	0,00	-509,69	-657,84	-361,55	0,00	Mean difference
Cream cheese (g/month)	-0,47	-20,94	19,99	0,96	-12,33	-31,90	7,25	0,22	-34,29	-60,09	-8,50	0,01	Mean difference
Natural yoghurt (g/month)	-259,68	-401,62	-117,75	0,00	-400,21	-535,83	-264,58	0,00	-485,97	-667,35	-304,59	0,00	Mean difference
Flavoured yoghurt (g/month)	26,35	-51,20	103,89	0,51	-69,21	-143,18	4,76	0,07	-31,31	-130,23	67,60	0,54	Mean difference
Kefir (g/month)	76,13	28,02	124,23	0,00	-119,92	-166,13	-73,72	0,00	-136,57	-197,92	-75,21	0,00	Mean difference
Plant-based drink (dl/month)	-14,27	-20,93	-7,61	0,00	-13,70	-20,24	-7,17	0,00	-13,81	-21,93	-5,69	0,00	Mean difference
Fruits (g/month)	-324,19	-1328,27	679,90	0,53	-89,35	-1053,33	874,63	0,86	242,74	-1061,52	1547,00	0,72	Mean difference
Vegetable (g/month)	-840,54	-2254,10	573,02	0,24	385,97	-976,09	1748,03	0,58	1731,02	-66,47	3528,51	0,06	Mean difference
Coffee (cup/day)	0,19	-0,33	0,70	0,47	0,74	0,25	1,23	0,00	0,83	0,17	1,49	0,01	Mean difference
Coffee (black)	0,50	0,27	0,91	0,02	1,42	0,85	2,35	0,18	1,46	0,76	2,82	0,26	Odds ratio
Coffee with milk	1,22	0,79	1,88	0,38	0,77	0,50	1,18	0,23	0,75	0,43	1,33	0,33	Odds ratio
Coffee with plant-based drink	0,95	0,53	1,68	0,85	0,71	0,40	1,27	0,25	0,34	0,13	0,87	0,02	Odds ratio
Coffee with sugar	1,11	0,62	1,99	0,73	0,75	0,41	1,36	0,34	0,27	0,10	0,75	0,01	Odds ratio
Coffee with sweetener	1,21	0,64	2,30	0,56	0,16	0,06	0,40	0,00	0,23	0,08	0,69	0,01	Odds ratio
Coffee with honey	2,99	1,02	8,80	0,05	0,13	0,01	1,18	0,07	0,77	0,14	4,31	0,76	Odds ratio

Supplementary Table S5: Detailed statistics with the 3SD cutoff

Variable	Mensa Hungary				Mensa Germany				Mensa United Kingdom				Effect size type
	Effect size	95% CI-l	95% CI-u	p	Effect size	95% CI-l	95% CI-u	p	Effect size	95% CI-l	95% CI-u	p	
Weight (kg)	1,60	-1,89	5,10	0,37	3,56	0,19	6,92	0,04	-3,97	-8,47	0,54	0,08	Mean difference
Height (cm)	0,37	-1,22	1,95	0,65	3,08	1,56	4,61	0,00	-0,07	-2,11	1,97	0,95	Mean difference
BMI (kg/m^2)	0,33	-0,67	1,34	0,52	-0,01	-0,98	0,96	0,98	-0,88	-2,17	0,41	0,18	Mean difference
Smoke	0,64	0,32	1,28	0,21	0,32	0,15	0,69	0,00	0,29	0,10	0,89	0,03	Odds ratio
Food diary	1,12	0,56	2,20	0,75	0,36	0,16	0,81	0,01	0,31	0,10	1,02	0,05	Odds ratio
Special food	1,11	0,70	1,76	0,67	0,80	0,51	1,26	0,34	0,78	0,42	1,44	0,43	Odds ratio
Gluten-free	0,33	0,15	0,71	0,00	0,11	0,04	0,27	0,00	0,13	0,04	0,45	0,00	Odds ratio
Lactose-free	0,98	0,54	1,78	0,95	0,35	0,18	0,69	0,00	0,19	0,06	0,57	0,00	Odds ratio
Dairy-free	0,74	0,36	1,52	0,41	0,15	0,06	0,40	0,00	0,35	0,11	1,09	0,07	Odds ratio
Sugar-free	0,54	0,27	1,06	0,07	0,10	0,04	0,25	0,00	0,05	0,01	0,27	0,00	Odds ratio
Walnut (g/month)	-47,62	-102,38	7,14	0,09	15,14	-37,66	67,94	0,57	-80,94	-150,15	-11,74	0,02	Mean difference
Pecans (g/month)	-13,61	-28,29	1,07	0,07	0,59	-13,57	14,75	0,93	-0,49	-19,01	18,03	0,96	Mean difference
Almond (g/month)	-28,03	-94,08	38,02	0,41	26,26	-37,37	89,89	0,42	30,57	-52,96	114,10	0,47	Mean difference
Sesame seed (g/month)	-5,81	-22,14	10,52	0,49	1,79	-13,97	17,56	0,82	-9,35	-29,98	11,27	0,37	Mean difference
Flaxseed (g/month)	-0,74	-38,00	36,53	0,97	84,85	49,01	120,69	0,00	-12,30	-59,59	35,00	0,61	Mean difference
Pumpkin seed	26,54	-24,16	77,23	0,31	15,27	-33,28	63,83	0,54	6,60	-57,10	70,31	0,84	Mean difference
Pine nut (g/month)	0,27	-7,23	7,77	0,94	15,36	8,12	22,59	0,00	12,58	3,13	22,04	0,01	Mean difference
Peanuts (g/month)	34,44	-22,21	91,10	0,23	78,65	24,04	133,26	0,00	31,67	-40,09	103,43	0,39	Mean difference
Sunflower seed	4,81	-32,21	41,84	0,80	43,48	8,02	78,95	0,02	-6,73	-53,39	39,94	0,78	Mean difference
Pistachio (g/month)	-9,75	-37,95	18,46	0,50	-34,94	-62,12	-7,77	0,01	-19,27	-54,94	16,40	0,29	Mean difference
Chia seed (g/month)	3,03	-23,61	29,67	0,82	-1,24	-27,01	24,53	0,92	1,94	-31,73	35,62	0,91	Mean difference
Poppy seed (g/month)	11,31	0,38	22,23	0,04	-9,93	-20,37	0,51	0,06	-14,44	-28,17	-0,72	0,04	Mean difference
Oil seeds (g/month)	193,12	-64,28	450,53	0,14	447,15	199,17	695,13	0,00	108,12	-214,33	430,57	0,51	Mean difference
Egg (piece/month)	-4,39	-8,49	-0,30	0,04	-13,19	-17,10	-9,27	0,00	-9,57	-14,73	-4,42	0,00	Mean difference
Meat products	314,62	10,15	619,09	0,04	-392,42	-694,61	-90,24	0,01	37,13	-380,20	454,46	0,86	Mean difference
Meat (g/month)	737,94	213,04	1262,83	0,01	-907,28	-1426,99	-387,58	0,00	685,66	-11,36	1382,69	0,05	Mean difference
Fish (g/month)	-102,95	-218,63	12,73	0,08	-57,12	-171,45	57,22	0,33	233,95	77,20	390,69	0,00	Mean difference
Sea food (g/month)	58,05	-16,23	132,33	0,13	65,93	-2,17	134,03	0,06	245,39	155,36	335,43	0,00	Mean difference
Chicken liver (g/month)	40,85	8,02	73,68	0,02	-42,51	-80,79	-4,23	0,03	-18,86	-91,53	53,80	0,61	Mean difference
Goose liver (g/month)	15,75	3,07	28,44	0,02	-0,88	-15,74	13,97	0,91	-8,90	-36,71	18,92	0,53	Mean difference
Milk <2% (dl/month)	-3,07	-9,98	3,84	0,38	-9,07	-15,74	-2,39	0,01	6,34	-2,60	15,29	0,16	Mean difference
Milk >2% (dl/month)	6,12	0,76	11,49	0,03	0,58	-4,61	5,77	0,83	0,02	-6,84	6,88	1,00	Mean difference
Sour cream <15% (g/month)	3,09	-11,12	17,29	0,67	-35,19	-48,74	-21,64	0,00	-47,82	-65,75	-29,89	0,00	Mean difference
Sour cream >15% (g/month)	51,92	35,01	68,84	0,00	-8,37	-24,48	7,74	0,31	-26,79	-48,25	-5,34	0,01	Mean difference
Cream (g/month)	0,42	-0,19	1,03	0,18	1,17	0,55	1,79	0,00	1,10	0,20	2,00	0,02	Mean difference
Cottage cheese	109,39	52,97	165,81	0,00	106,16	51,44	160,88	0,00	-68,27	-140,37	3,83	0,06	Mean difference
Cheese (g/month)	110,64	-18,72	239,99	0,09	-355,95	-479,71	-232,19	0,00	-561,38	-725,25	-397,52	0,00	Mean difference
Cream cheese	-20,65	-47,39	6,09	0,13	-36,03	-61,66	-10,39	0,01	-56,86	-90,79	-22,93	0,00	Mean difference
Natural yoghurt	-260,09	-508,00	-12,18	0,04	-544,42	-784,14	-304,69	0,00	-644,87	-965,19	-324,55	0,00	Mean difference
Flavoured yoghurt (g/month)	-12,58	-111,85	86,70	0,80	-156,83	-251,96	-61,69	0,00	-105,43	-233,03	22,17	0,11	Mean difference
Kefir (g/month)	76,44	21,90	130,99	0,01	-131,23	-183,68	-78,77	0,00	-146,62	-216,31	-76,92	0,00	Mean difference
Plant-based drink (dl/month)	-14,16	-21,62	-6,70	0,00	-10,61	-17,90	-3,32	0,00	-13,19	-22,27	-4,10	0,00	Mean difference
Fruits (g/month)	-645,86	-1965,32	673,61	0,34	45,53	-1215,44	1306,50	0,94	-199,21	-1916,69	1518,28	0,82	Mean difference
Vegetable (g/month)	-840,54	-2254,10	573,02	0,24	385,97	-976,09	1748,03	0,58	1731,02	-66,47	3528,51	0,06	Mean difference
Coffee (cup/day)	0,19	-0,33	0,70	0,47	0,74	0,25	1,23	0,00	0,83	0,17	1,49	0,01	Mean difference
Coffee (black)	0,50	0,27	0,91	0,02	1,42	0,85	2,35	0,18	1,46	0,76	2,82	0,26	Odds ratio
Coffee with milk	1,22	0,79	1,88	0,38	0,77	0,50	1,18	0,23	0,75	0,43	1,33	0,33	Odds ratio
Coffee with plant-based drink	0,95	0,53	1,68	0,85	0,71	0,40	1,27	0,25	0,34	0,13	0,87	0,02	Odds ratio
Coffee with sugar	1,11	0,62	1,99	0,73	0,75	0,41	1,36	0,34	0,27	0,10	0,75	0,01	Odds ratio
Coffee with sweetener	1,21	0,64	2,30	0,56	0,16	0,06	0,40	0,00	0,23	0,08	0,69	0,01	Odds ratio
Coffee with honey	2,99	1,02	8,80	0,05	0,13	0,01	1,18	0,07	0,77	0,14	4,31	0,76	Odds ratio

Supplementary Table S6: Detailed statistics without outlier filtering

Variable	Mensa Hungary				Mensa Germany				Mensa United Kingdom				Effect size type
	Effect size	95% CI-l	95% CI-u	p	Effect size	95% CI-l	95% CI-u	p	Effect size	95% CI-l	95% CI-u	p	
Weight (kg)	1,21	-2,46	4,89	0,52	3,14	-0,38	6,67	0,08	-3,63	-8,34	1,07	0,13	Mean difference
Height (cm)	0,36	-1,24	1,95	0,66	3,17	1,64	4,71	0,00	-0,08	-2,13	1,97	0,94	Mean difference
BMI (kg/m^2)	0,26	-0,88	1,40	0,65	0,11	-0,99	1,20	0,85	-1,21	-2,67	0,25	0,10	Mean difference
Smoke	0,64	0,32	1,28	0,21	0,32	0,15	0,69	0,00	0,29	0,10	0,89	0,03	Odds ratio
Food diary	1,12	0,56	2,20	0,75	0,36	0,16	0,81	0,01	0,31	0,10	1,02	0,05	Odds ratio
Special food	1,11	0,70	1,76	0,67	0,80	0,51	1,26	0,34	0,78	0,42	1,44	0,43	Odds ratio
Gluten-free	0,33	0,15	0,71	0,00	0,11	0,04	0,27	0,00	0,13	0,04	0,45	0,00	Odds ratio
Lactose-free	0,98	0,54	1,78	0,95	0,35	0,18	0,69	0,00	0,19	0,06	0,57	0,00	Odds ratio
Dairy-free	0,74	0,36	1,52	0,41	0,15	0,06	0,40	0,00	0,35	0,11	1,09	0,07	Odds ratio
Sugar-free	0,54	0,27	1,06	0,07	0,10	0,04	0,25	0,00	0,05	0,01	0,27	0,00	Odds ratio
Walnut (g/month)	-152,93	-315,27	9,40	0,07	-58,89	-215,17	97,38	0,46	-197,11	-402,34	8,11	0,06	Mean difference
Pecans (g/month)	-25,15	-57,34	7,03	0,13	-0,11	-31,16	30,94	0,99	-13,04	-53,77	27,69	0,53	Mean difference
Almond (g/month)	-136,98	-302,89	28,94	0,11	-78,21	-237,94	81,51	0,34	-104,78	-314,98	105,42	0,33	Mean difference
Sesame seed (g/month)	-13,81	-45,08	17,45	0,39	8,82	-21,28	38,91	0,57	-18,89	-58,49	20,70	0,35	Mean difference
Flaxseed (g/month)	-3,14	-51,94	45,67	0,90	80,96	33,96	127,96	0,00	-8,73	-70,71	53,26	0,78	Mean difference
Pumpkin seed (g/month)	164,91	1,40	328,41	0,05	-3,86	-161,25	153,52	0,96	-28,91	-235,28	177,46	0,78	Mean difference
Pine nut (g/month)	-3,39	-17,02	10,24	0,63	20,36	7,23	33,49	0,00	9,34	-7,88	26,55	0,29	Mean difference
Peanuts (g/month)	28,79	-111,83	169,41	0,69	67,88	-67,42	203,18	0,33	11,86	-166,42	190,14	0,90	Mean difference
Sunflower seed (g/month)	44,14	-33,60	121,87	0,27	46,27	-28,54	121,09	0,23	18,42	-79,93	116,76	0,71	Mean difference
Pistachio (g/month)	-51,68	-139,22	35,86	0,25	-76,43	-160,81	7,96	0,08	-85,86	-196,86	25,14	0,13	Mean difference
Chia seed (g/month)	-54,22	-106,92	-1,53	0,04	-40,35	-91,07	10,37	0,12	-34,20	-101,02	32,61	0,32	Mean difference
Poppy seed (g/month)	11,43	-10,35	33,21	0,30	-28,61	-49,59	-7,64	0,01	-23,95	-51,49	3,59	0,09	Mean difference
Oil seeds (g/month)	-39,73	-514,02	434,56	0,87	89,14	-367,88	546,16	0,70	-320,20	-916,01	275,60	0,29	Mean difference
Egg (piece/month)	-1,99	-8,56	4,58	0,55	-12,96	-19,29	-6,64	0,00	-9,71	-18,08	-1,33	0,02	Mean difference
Meat products (g/month)	305,46	-97,21	708,13	0,14	-471,05	-872,26	-69,84	0,02	30,57	-518,29	579,43	0,91	Mean difference
Meat (g/month)	651,45	75,42	1227,48	0,03	-924,66	-1493,06	-356,27	0,00	616,34	-151,37	1384,05	0,12	Mean difference
Fish (g/month)	-114,98	-271,90	41,95	0,15	-46,72	-201,56	108,12	0,55	411,57	202,43	620,72	0,00	Mean difference
Sea food (g/month)	41,37	-59,13	141,87	0,42	43,33	-49,29	135,96	0,36	280,33	160,26	400,39	0,00	Mean difference
Chicken liver (g/month)	34,97	-21,49	91,43	0,23	-51,45	-117,42	14,51	0,13	31,70	-92,32	155,71	0,62	Mean difference
Goose liver (g/month)	0,38	-27,05	27,82	0,98	-1,31	-33,37	30,75	0,94	-20,13	-80,39	40,14	0,51	Mean difference
Milk <2% (dl/month)	-8,61	-18,97	1,75	0,10	-13,28	-23,27	-3,30	0,01	1,83	-11,56	15,22	0,79	Mean difference
Milk >2% (dl/month)	7,82	-0,87	16,50	0,08	-3,74	-12,17	4,69	0,38	-3,31	-14,43	7,81	0,56	Mean difference
Sour cream <15% (g/month)	9,76	-20,09	39,61	0,52	-53,71	-82,41	-25,00	0,00	-66,99	-105,07	-28,90	0,00	Mean difference
Sour cream >15% (g/month)	53,14	16,53	89,74	0,00	-56,66	-91,82	-21,49	0,00	-88,11	-135,00	-41,21	0,00	Mean difference
Cream (g/month)	-0,87	-2,47	0,73	0,29	-0,08	-1,70	1,55	0,93	0,41	-1,95	2,77	0,73	Mean difference
Cottage cheese (g/month)	11,23	-126,16	148,61	0,87	72,92	-60,22	206,05	0,28	-224,56	-400,58	-48,54	0,01	Mean difference
Cheese (g/month)	149,29	-49,81	348,40	0,14	-417,89	-609,17	-226,60	0,00	-734,60	-988,78	-480,41	0,00	Mean difference
Cream cheese (g/month)	-43,03	-98,93	12,86	0,13	-81,20	-135,14	-27,26	0,00	-125,82	-197,38	-54,26	0,00	Mean difference
Natural yoghurt (g/month)	-148,68	-480,04	182,69	0,38	-614,71	-936,91	-292,51	0,00	-680,96	-1111,26	-250,67	0,00	Mean difference
Flavoured yoghurt (g/month)	-144,75	-344,56	55,07	0,16	-399,70	-592,46	-206,93	0,00	-314,11	-572,85	-55,37	0,02	Mean difference

Variable	Mensa Hungary				Mensa Germany				Mensa United Kingdom				Effect size type
	Effect size	95% CI-l	95% CI-u	p	Effect size	95% CI-l	95% CI-u	p	Effect size	95% CI-l	95% CI-u	p	
Kefir (g/month)	2,54	-163,48	168,56	0,98	-281,00	-440,84	-121,16	0,00	-250,39	-462,29	-38,50	0,02	Mean difference
Plant-based drink (dl/month)	-43,15	-56,25	-30,05	0,00	-37,81	-50,58	-25,04	0,00	-35,33	-51,33	-19,34	0,00	Mean difference
Fruits (g/month)	-1167,90	-2768,02	432,21	0,15	-341,10	-1869,71	1187,50	0,66	822,42	-1238,37	2883,21	0,43	Mean difference
Vegetable (g/month)	-1008,39	-2560,11	543,34	0,20	311,39	-1181,31	1804,09	0,68	1315,25	-665,01	3295,50	0,19	Mean difference
Coffee (cup/day)	0,19	-0,33	0,70	0,47	0,74	0,25	1,23	0,00	0,83	0,17	1,49	0,01	Mean difference
Coffee (black)	0,50	0,27	0,91	0,02	1,42	0,85	2,35	0,18	1,46	0,76	2,82	0,26	Odds ratio
Coffee with milk	1,22	0,79	1,88	0,38	0,77	0,50	1,18	0,23	0,75	0,43	1,33	0,33	Odds ratio
Coffee with plant-based drink	0,95	0,53	1,68	0,85	0,71	0,40	1,27	0,25	0,34	0,13	0,87	0,02	Odds ratio
Coffee with sugar	1,11	0,62	1,99	0,73	0,75	0,41	1,36	0,34	0,27	0,10	0,75	0,01	Odds ratio
Coffee with sweetener	1,21	0,64	2,30	0,56	0,16	0,06	0,40	0,00	0,23	0,08	0,69	0,01	Odds ratio
Coffee with honey	2,99	1,02	8,80	0,05	0,13	0,01	1,18	0,07	0,77	0,14	4,31	0,76	Odds ratio

Supplementary Table S7: Comparison of effect sizes and p-values before and after controlling for years of education for across-country replicated variables

Variable	Before controlling for years of education						After controlling for years of education						Effect size type
	Mensa Hungary		Mensa Germany		Mensa United Kingdom		Mensa Hungary		Mensa Germany		Mensa United Kingdom		
	Effect size	p	Effect size	p	Effect size	p	Effect size	p	Effect size	p	Effect size	p	
Gluten-free	0,331	0,004	0,106	0,000	0,132	0,448	0,311	0,004	0,113	0,000	0,146	0,001	Odds ratio
Lactose-free	0,981	0,951	0,354	0,002	0,191	0,574	1,020	0,950	0,385	0,005	0,191	0,003	Odds ratio
Sugar-free	0,535	0,073	0,097	0,000	0,054	0,272	0,616	0,184	0,128	0,000	0,056	0,000	Odds ratio
Cream (g/month)	0,343	0,222	1,047	0,000	1,186	0,004	0,425	0,136	1,116	0,000	1,183	0,004	Mean difference
Natural yoghurt (g/month)	-259,683	0,000	-400,206	0,000	-485,971	0,000	-282,036	0,000	-418,207	0,000	-495,490	0,000	Mean difference
Plant-based drink (dl/month)	-14,269	0,000	-13,704	0,000	-13,809	0,001	-14,448	0,000	-13,851	0,000	-13,891	0,001	Mean difference
Egg (piece/month)	-4,312	0,028	-12,206	0,000	-9,498	-4,666	-4,262	0,019	-11,182	0,000	-8,259	0,000	Mean difference
Coffee (cup/day)	0,188	0,474	0,740	0,003	0,833	0,013	0,161	0,544	0,717	0,005	0,822	0,015	Mean difference
Coffee with sweetener	1,211	0,558	0,159	0,000	0,233	0,008	1,433	0,284	0,177	0,000	0,242	0,011	Odds ratio

Supplementary Table S8: Comparison of effect sizes and p-values in the original and Hungarian-only analyses for across-country replicated variables

Variable	Mensa Hungary original				Mensa Hungary Hungarian-only				Effect size type
	Effect size	95% CI-l	95% CI-u	p	Effect size	95% CI-l	95% CI-u	p	
Gluten-free	0,33	0,15	0,71	0,004	0,40	0,18	0,86	0,020	Odds ratio
Lactose-free	0,98	0,54	1,78	0,951	1,24	0,66	2,33	0,497	Odds ratio
Sugar-free	0,54	0,27	1,06	0,073	0,57	0,29	1,14	0,115	Odds ratio
Cream (g/month)	0,34	-0,21	0,89	0,222	0,07	-0,58	0,73	0,821	Mean difference
Natural yoghurt (g/month)	-259,68	-401,62	-117,75	0,000	-173,04	-315,21	-30,87	0,017	Mean difference
Plant-based drink (dl/month)	-14,27	-20,93	-7,61	0,000	-17,35	-23,99	-10,70	0,000	Mean difference
Egg (piece/month)	-4,31	-8,15	-0,48	0,028	-2,67	-6,52	1,17	0,174	Mean difference
Coffee (cup/day)	0,19	-0,33	0,70	0,474	0,432	-0,15	1,01	0,143	Mean difference
Coffee with sweetener	1,21	0,64	2,30	0,558	1,08	0,42	1,46	0,833	Odds ratio

Supplementary Table S9: Comparison of effect sizes and p-values in national Mensa vs. control group analyses using international and Hungarian-only controls

Variable	Mensa Hungary Hungarian-only				Pooled Mensa				Effect size type
	Effect size	95% CI-l	95% CI-u	p	Effect size	95% CI-l	95% CI-u	p	
Gluten-free	0,40	0,18	0,86	0,020	4,94	2,47	9,88	0,000	Odds ratio
Lactose-free	1,24	0,66	2,33	0,497	1,68	0,97	2,92	0,065	Odds ratio
Sugar-free	0,57	0,29	1,14	0,115	3,76	1,96	7,20	0,000	Odds ratio
Cream (g/month)	0,07	-0,58	0,73	0,821	-0,69	-1,19	-0,19	0,007	Mean difference
Natural yoghurt (g/month)	-173,04	-315,21	-30,87	0,017	343,64	219,3	467,97	0,000	Mean difference
Plant-based drink (dl/month)	-17,35	-23,99	-10,70	0,000	13,97	7,80	20,13	0,000	Mean difference
Egg (piece/month)	-2,67	-6,52	1,17	0,174	8,21	5,11	11,31	0,000	Mean difference
Coffee (cup/day)	0,432	-0,15	1,01	0,143	-0,50	-0,95	-0,05	0,030	Mean difference
Coffee with sweetener	1,08	0,42	1,46	0,833	1,74	0,93	3,25	0,085	Odds ratio
